# Increasing cell biomass in *Saccharomyces cerevisiae *increases recombinant protein yield: the use of a respiratory strain as a microbial cell factory

**DOI:** 10.1186/1475-2859-9-47

**Published:** 2010-06-17

**Authors:** Cecilia Ferndahl, Nicklas Bonander, Christel Logez, Renaud Wagner, Lena Gustafsson, Christer Larsson, Kristina Hedfalk, Richard AJ Darby, Roslyn M Bill

**Affiliations:** 1 Chemical and Biological Engineering/Molecular Biotechnology, Chalmers University of Technology, 412 96 Göteborg, Sweden; 2 School of Life and Health Sciences, Aston University, Aston Triangle, Birmingham B4 7ET, UK; 3 UMR 7175 - LC1, Dpt Récepteurs et Protéines Membranaires, ESBS, Blvd Sébastien Brant, BP 10413, 67412 Illkirch Cedex, France; 4 Department of Chemistry/Biochemistry, Göteborg University, Box 462, 405 30 Göteborg, Sweden

## Abstract

**Background:**

Recombinant protein production is universally employed as a solution to obtain the milligram to gram quantities of a given protein required for applications as diverse as structural genomics and biopharmaceutical manufacture. Yeast is a well-established recombinant host cell for these purposes. In this study we wanted to investigate whether our respiratory *Saccharomyces cerevisiae *strain, TM6*, could be used to enhance the productivity of recombinant proteins over that obtained from corresponding wild type, respiro-fermentative strains when cultured under the same laboratory conditions.

**Results:**

Here we demonstrate at least a doubling in productivity over wild-type strains for three recombinant membrane proteins and one recombinant soluble protein produced in TM6* cells. In all cases, this was attributed to the improved biomass properties of the strain. The yield profile across the growth curve was also more stable than in a wild-type strain, and was not further improved by lowering culture temperatures. This has the added benefit that improved yields can be attained rapidly at the yeast's optimal growth conditions. Importantly, improved productivity could not be reproduced in wild-type strains by culturing them under glucose fed-batch conditions: despite having achieved very similar biomass yields to those achieved by TM6* cultures, the total volumetric yields were not concomitantly increased. Furthermore, the productivity of TM6* was unaffected by growing cultures in the presence of ethanol. These findings support the unique properties of TM6* as a microbial cell factory.

**Conclusions:**

The accumulation of biomass in yeast cell factories is not necessarily correlated with a proportional increase in the functional yield of the recombinant protein being produced. The respiratory *S. cerevisiae *strain reported here is therefore a useful addition to the matrix of production hosts currently available as its improved biomass properties do lead to increased volumetric yields without the need to resort to complex control or cultivation schemes. This is anticipated to be of particular value in the production of challenging targets such as membrane proteins.

## Background

The development of recombinant protein production systems that can be applied to a wide range of targets is a key area of research. This is particularly true for the production of membrane proteins, which are high value targets in the drug discovery pipeline, and which cannot yet be produced in high yields in a predictable manner. Grisshammer and Tate first addressed these issues in 1995 in their review of the challenges of producing recombinant membrane proteins [1]. While more recent articles have individually covered the use of bacteria [2-5], yeasts [6], insect cells [7], mammalian cells [8] and cell-free systems [9] as production hosts [10,11], it is apparent that generic solutions are still not forthcoming and that the way forward should be through a rationalization of the science of protein production [12-14].

Yeast species, especially *Pichia pastoris *and *S. cerevisiae *[6,11,15,16] have already been identified as one of the most important components of a matrix of protein production hosts [12], and have contributed a substantial number of recombinant eukaryotic membrane proteins that have enabled high resolution structure determination [17-20]. We and others [21-23] have recently started to take a more systematic approach [14] to optimise *S. cerevisiae *as a host cell for recombinant protein production. In general, a sequenced genome and well-understood expression vectors with a range of promoters and expression tags make this yeast species an attractive and flexible option. While the *P. pastoris *system has a much smaller set of vectors available and a less-well-established molecular biology, it can be cultured to very high densities [24] potentially producing large quantities of the protein of interest. Recently we examined factors, including pre-induction cellular biomass, affecting the total yield of recombinant green fluorescent protein (GFP) in *P. pastoris *[25], and noted the benefits of this as a strategy to improve productivity. While this approach is generally accepted to be a useful way to boost the yields of soluble proteins, it is well established that the accumulation of biomass does not necessarily lead to a correlated increase in membrane protein yield [21] and in the case of G protein-coupled receptors (GPCRs), specific activity is often lower [26]. Indeed, it has been noted that higher cell densities can generate cellular stresses leading to modifications in membrane composition [27] and that this modified environment influences the activity of recombinant proteins. Consequently, medium cell density fermentation procedures for GPCR expression in *P. pastoris *have been suggested to be preferable to ones where biomass yields are maximised [26].

Here we examined a respiratory strain of *S. cerevisiae*, TM6*, which generates substantially higher biomass yields than wild-type at the expense of ethanol formation [28,29]. We asked whether this strain could be used as a tool to generate improved volumetric yields of functional proteins, especially membrane proteins. Since this strain has an engineered phenotype, we anticipated that this might mitigate against the cellular stress associated with biomass accumulation in wild-type strains. In the TM6* strain, glucose uptake is solely dependent on a chimeric hexose transporter mediating reduced sugar uptake: the strain was generated by integrating the gene encoding the chimeric hexose transporter, Tm6*, into the genome of a hexose transporter null yeast. While oxygen depletion commonly controls the switch from respiration to fermentation, in wild-type *S. cerevisiae *this switch also occurs in response to the external glucose concentration. The TM6* strain shows a fully respiratory metabolism even at high glucose levels as seen for aerobic organisms due to the characteristics of its chimeric hexose transporter. It therefore switches to fermentation only when oxygen is limiting.

In this study, the effectiveness of the TM6* strain as a microbial cell factory was tested using three challenging membrane protein targets: the eukaryotic glycerol transport facilitator, Fps1 [30,31] and two recombinant human GPCRs (the A_2a _adenosine receptor (A_2a_R) and the cannabinoid receptor 2 (CNR2)); as well as soluble GFP. Our start point was to examine TM6* under conditions that we had previously shown to give maximum recombinant Fps1 yield for the corresponding wild-type [21] and to compare this with the yield obtained under optimal growth conditions. Therefore TM6* cells were cultured at 20°C, pH 5 and 30°C, pH 5, respectively. Our data show at least a doubling in productivity over wild-type strains for all these proteins when they are produced in TM6* cells. In all cases, this was attributed to the improved biomass properties of the strain. The yield profile across the growth curve was also found to be more stable than in the wild-type strain, and was not further improved by lowering culture temperatures to 20°C, which is commonly found for wild-type strains [21]. This has the added benefit that improved yields can be attained rapidly at the yeast's optimal growth conditions. This improved productivity could not simply be reproduced in a wild-type strain by culturing it under glucose fed-batch conditions prior to glucose exhaustion. Likewise, the productivity of TM6* was unaffected by culturing it in the presence of ethanol.

To conclude, the beneficial properties of TM6* appear to be unique. Consequently, TM6* should prove to be a useful addition to the matrix of production hosts at the disposal of modern structural biology projects, especially for challenging targets such as membrane proteins: we suggest that transfer of expression plasmids from wild-type strains into TM6* is a simple way to at least double the yield of a range of recombinant proteins.

## Results

We have previously demonstrated that Fps1 is a challenging eukaryotic membrane protein target that can only be produced in low yields in a variety of hosts (Table 1). Subsequently, we demonstrated that for wild-type *S. cerevisiae *the best yields were achieved when the culture temperature was decreased from 30°C to 20°C and the cells were harvested at a precise time point [21]. In this study, we explored whether increasing the biomass yield of *S. cerevisiae *cultures by simply switching to the respiratory TM6* strain under the same culture conditions could be an alternative strategy to improve productivity.

**Table 1:** Fps1 can be produced in a range of microbial cell factories

Production Host	Promoter	Production	Localisation	Reference
*S. cerevisiae *W301-1A	*GAL1*	Yes	tm	[38]
*S. cerevisiae *W301-1A	*TPI1*	Yes	tm	[38]
*S. cerevisiae *954 VW K70	*TPI1*	Yes	tm	[21] and this study
*S. cerevisiae *KOY.PK2-1C82	*TPI1*	Yes	tm	This study
*S. cerevisiae *TM6*	*TPI1*	Yes	tm	This study
*P. pastoris *X-33	*AOX1*	Yes	pm	[38]
*P. pastoris *X-33	*AOX1*	Yes^†^	nd	[38]
*P. pastoris *GS115	*AOX1*	Yes	nd	[38]
*P. pastoris *KM71	*AOX1*	Yes	nd	[38]
*E. coli *JM109	lac	Yes	tm	[38]
*E. coli *JM109	T7/lac	Yes	tm	[38]

Fps1 was produced in *S. cerevisiae*, *P. pastoris *and *E. coli *as previously described [21,38] and as a part of this study (tm, total membrane; pm, plasma membrane; nd, not determined). Note that ^† ^indicates that the *α*-factor signal peptide was used in the expression construct. In all cases prior to this study, total yields were less than 50 μg/L, which was below the threshold value for further study [21,38].

### Fps1 can be produced in TM6* at the same yield per unit of total membrane protein as two wild-type strains

We analysed the production of recombinant Fps1 in TM6*, which has improved biomass production properties [29], and compared it to production under identical culture regimes in two typical wild-type strains: the wild-type, respiro-fermentative 954 VW K70 and KOY.PK2-1C82 strains of *S. cerevisiae*. The former strain was used in our previous study [21] and the latter is derived from CEN.PK2-1C, which is the genetic background of TM6*. The yield of Fps1 per unit of total membrane protein was quantified by densitometry of immunoblot signals from 75 μg total membrane protein. For quantifying Fps1 in crude cell extracts, signals from 35 μg total cell extract protein were used. All signals were normalised to our previously-described internal standard [21]. Table 2 shows that when cultured at 30°C, pH5, all three hosts gave the same average yield per unit of total membrane protein.

**Table 2:** Recombinant Fps1 yields per unit of total membrane protein are identical for two wild-type *S. cerevisiae *strains and respiratory TM6*

	Fps1 yield expressed as a mean densitometry signal			
Strains	All samples	Glucose phase	Ethanol phase	Stationary phase
954 VW K70	3.3 (0.3; n = 2)	3.2 (0.1; n = 2)	3.5 (0.9; n = 2)	2.6 (0.8; n = 2)
KOY.PK2-1C82	3.7 (0.6; n = 3)	3.7 (0.6; n = 3)	3.7 (0.9; n = 3)	2.5 (0.9; n = 3)
TM6*	3.1 (0.7; n = 3)	3.2 (0.8; n = 3)	-	2.4 (0.9; n = 3)

Immunoblots were performed and quantified on membrane-bound fractions from two wild-type cultures and the TM6* strain, producing recombinant Fps1. The values given are mean densitometry signals related to our internal standard as described in the Methods section. Standard error of the mean, in parentheses, was calculated for replicate cultures as indicated. Note that there is no significant yield improvement per unit of total membrane protein for production in TM6* compared with these wild-types: increased yield is achieved on account of improved biomass production in TM6* (see Figure 2). Note also that since TM6* does not have an ethanol phase (-), the "stationary phase" samples were harvested at least 10 h after glucose exhaustion.

**Figure 1:**
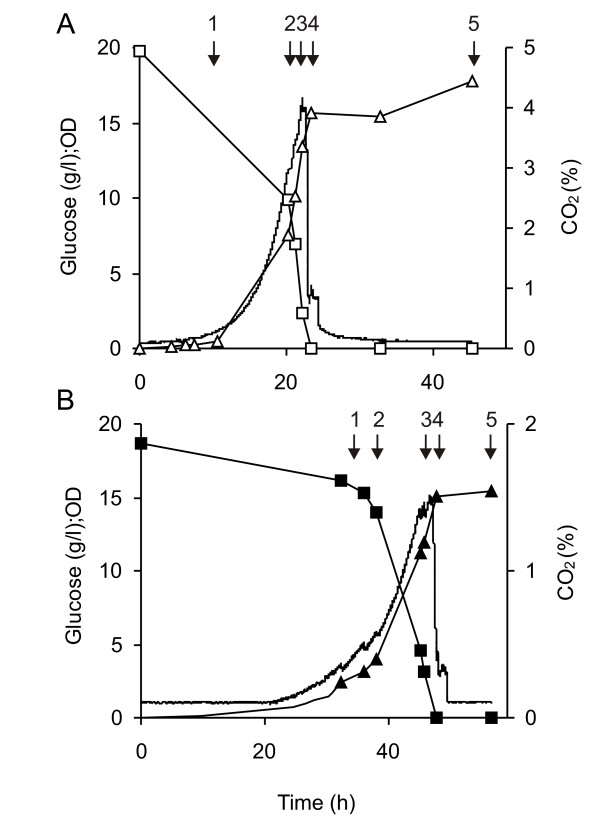
Culture profiles for TM6* transformants grown aerobically on 2 % glucose. Cells were cultured at (A) 30°C, pH 5 and (B) 20°C, pH 5. Glucose concentration (squares), OD_610 _(triangles) and CO_2 _level (no symbol) versus time is shown for growth under these conditions. The sampling points are indicated with an arrow, and each sample is also given a number for ease of identification.

On reducing the temperature of the recombinant TM6* cultures to 20°C, we noted no statistically significant improvement in their productivity (data not shown). This is in contrast to the commonly-observed increase in yield seen on lowering culture temperatures in wild-type yeast [21,32], as well as other recombinant hosts [14,33]. The gas profiles (Figure 1) were similar for both 20°C, pH5 and 30°C, pH5 and showed only one phase of growth: the small amounts of ethanol produced did not result in a biphasic growth curve. The generation times calculated under these conditions were 5.5 (standard error of the mean, SEM: 0.5) h and 2.9 (0.1) h at 20°C and 30°C, respectively. The duration of the glucose phase was significantly longer for the 20°C culture (64.3 (9.5) h) compared with that for 30°C (27.0 (2.9) h), as expected. These temperature-dependent data are consistent with our previously-reported growth characteristics for a wild-type strain [21]. We also noted that the lag phase for the 20°C cultures was much more variable than for the 30°C cultures (data not shown). Ethanol analysis revealed a maximum yield of 0.03 (0.02) g g^-1 ^consumed glucose at 20°C and 0.07 (0.02) g g^-1 ^at 30°C, compared with a typical value of 0.3 g g^-1 ^for a wild-type strain at 30°C. The maximum glucose consumption rate was more than doubled on changing the temperature from 20°C to 30°C with rates of 2.0 (0.3) and 5.1 (0.6) mmol (glucose) g^-1 ^(dry weight) h^-1^, respectively.

### Total volumetric yields of Fps1 from wild-type strains can be more than doubled in TM6* due to its improved biomass yield

**Figure 2:**
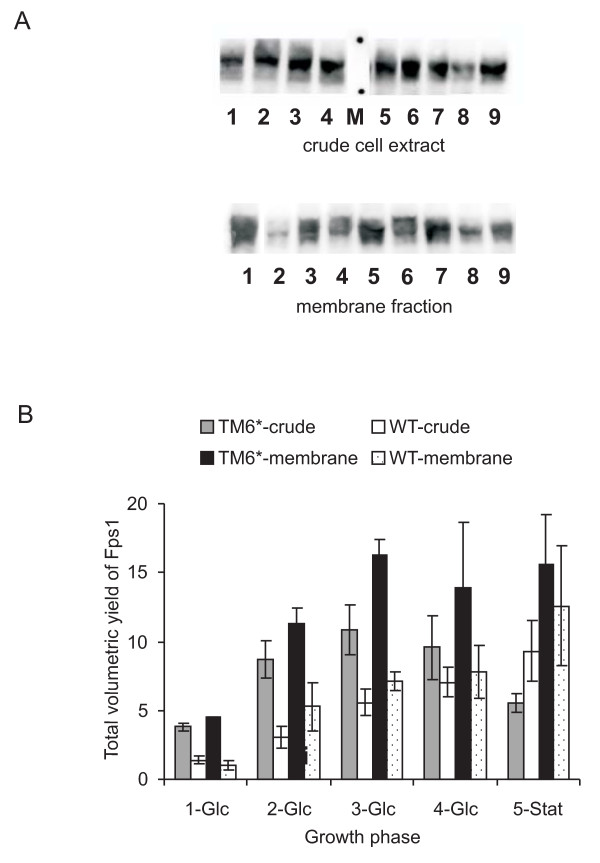
Fps1 yields produced from KOY.PK2-1C82 and TM6* cells. Samples were harvested at comparable residual glucose concentrations as indicated in Figure 1. (A) Immunoblots of crude cellular extracts (upper panel) of Fps1 produced in wild-type KOY.PK2-1C82 (lanes 1-4) and TM6* cells (lanes 5-8) are shown. Lane 9 is the internal standard. Lanes 1-4 and 5-8 correspond to 1-Glc, 2-Glc, 3-Glc and 4-Glc, in Figure 2B. The marker (M) lane highlights the 98 and 62 kDa standards. The corresponding membrane fraction is shown in the lower panel. Wild-type cells are lanes 2, 4, 6 and 8, and TM6* cells are lanes 3, 5, 7 and 9. Lane 1 is the internal standard. (B) The Fps1 yield per unit of protein was derived from immunoblots such as those shown in Figure 2A and quantified relative to a standard for both the crude cell extract and membrane-bound fractions as described in the Methods section. The total volumetric yield was then derived by multiplying with the corresponding dry weights. Triplicate fermentations were used to calculate the standard error of the mean (SEM).

When the biomass of the cultures was taken into account, improved volumetric yields for TM6* were clearly apparent compared with wild-type cells (Figure 2). At 30°C, pH 5, yields per unit of total membrane protein from glucose-phase cells (at harvest points 1-3) showed more than a 2-fold increase when comparing TM6* with wild-type (mean values were 16.3 and 7.8 respectively; Figure 2B). This meant that the doubling in yield we had previously obtained by lowering the temperature of wild-type cultures from 30°C to 20°C [21] had now been exceeded by switching to the TM6* strain and culturing at 30°C. Essentially, the highest yields were now being produced under the optimal growth conditions: more Fps1 is produced more quickly in TM6* (generation time 2.9 h (SEM: 0.1)) than in the previously-optimised wild-type cultures at 20°C (generation time 8.9 h [21]).

### Improved Fps1 yields in TM6* cannot be reproduced by culturing wild-type strains under glucose fed-batch conditions prior to glucose exhaustion

**Figure 3:**
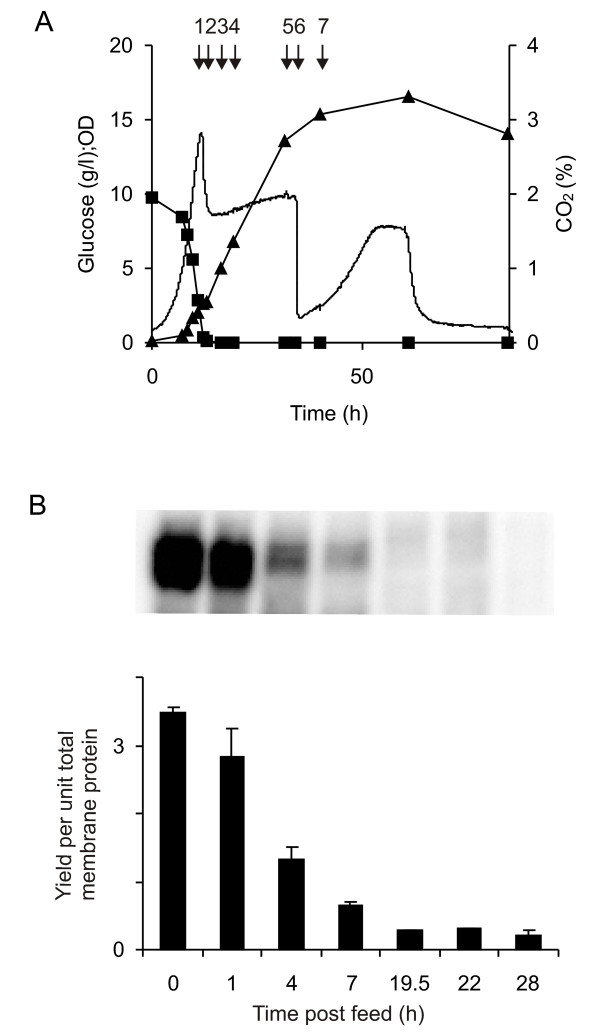
Analysis of protein yields for a wild-type strain grown under glucose fed-batch conditions in an attempt to mimic TM6*. (A) CO_2 _production (no symbol), glucose consumption (squares) and OD_610 _(triangles) show classic profiles for a fed-batch culture. Arrow 1 indicates the onset of the feed (0 h) and arrow 6 the end (22 h). (B) An immunoblot (upper panel) showing the Fps1 production at 0, 1, 4, 7, 19.5, 22 and 28 h after the onset of the feed: its quantitation (lower panel) is related to the internal standard.

In an attempt to improve biomass yields for the wild-type strain and thereby obtain improved volumetric yields as seen in the TM6* cultures, the wild-type strain 954 VW K70 was cultured at 30°C, pH 5 with 1 % glucose until the glucose was depleted and then grown under glucose fed-batch conditions. This was done in order to determine whether manipulating the culture regime to extend the glucose phase of the wild-type strain would produce higher amounts of Fps1. The substrate was fed to the culture at a rate of 2 g L^-1 ^h^-1 ^to a final concentration of 2 % glucose (w/v). Figure 3A shows the profile of CO_2 _production, glucose consumption and OD_610_, which was as anticipated: CO_2 _production is essentially constant during the glucose feed with the ethanol being respired immediately after the glucose feed is finished, which is seen from the second CO_2 _peak. Figure 3B shows that the yield of Fps1 per unit of total membrane protein was neither sustained nor increased during the fed-batch. On the contrary, the yield decreased with the onset of the fed-batch, indicating that by essentially extending the glucose growth phase, the total volumetric yield from wild-type cells was not improved: despite having achieved very similar biomass yields to those achieved by TM6* cultures (Figure 1), the total volumetric yields were not concomitantly increased. It was therefore clear that the simple manipulation of culture conditions for a wild-type strain could not mimic the properties of TM6*.

### Fps1 yields in TM6* are unaffected by growing cultures in the presence of ethanol

**Figure 4:**
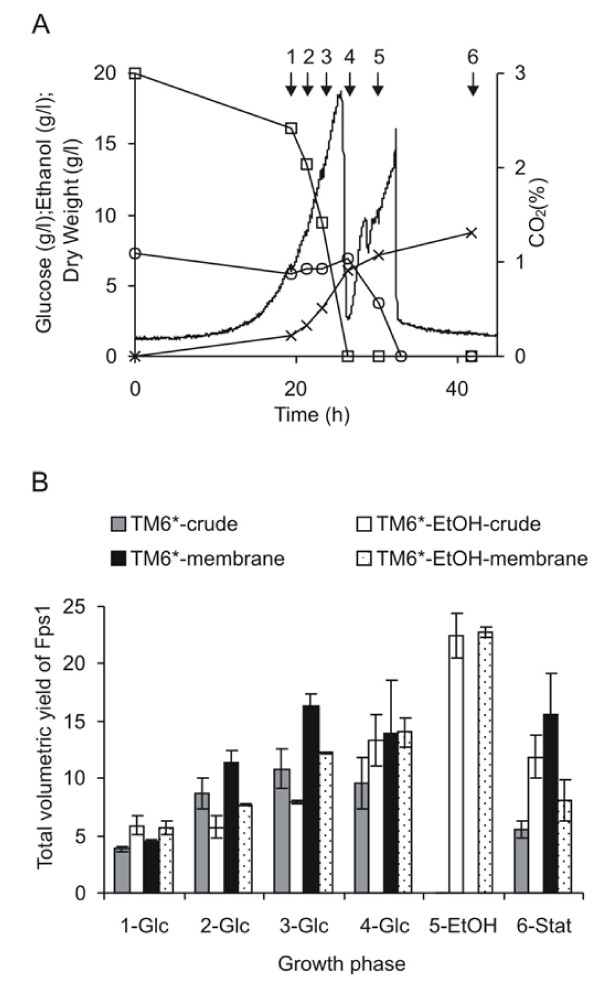
Analysis of protein yields for TM6* grown on 2 % glucose in the presence and absence of ethanol. (A) CO_2 _production (no symbol), glucose consumption (squares), ethanol concentration (circles) and dry weights (crosses) were measured. Samples were harvested at the time points indicated with arrows (given numbers for the ease of identification). (B) The yield of Fps1 was related to the appropriate dry weight for the time-point to give the total volumetric yield. At least duplicate fermentations were used to calculate the SEM.

We next investigated whether yields from respiratory TM6* cells would return to wild-type levels in the presence of ethanol. TM6* was therefore cultured at 30°C, pH 5 in the presence of 2 % glucose and 7 g L^-1 ^ethanol to simulate the levels observed in wild type strains. Figure 4A shows that the ethanol concentration was essentially constant through the glucose consumption phase, indicating that the TM6* cells grew on glucose without any substantial consumption of ethanol. At the point of glucose depletion, the cells started to grow on the ethanol immediately which is seen by the second CO_2 _peak and the sustained increase in biomass. The total volumetric yield of Fps1 in the cultures to which ethanol had been added was higher than those without ethanol on account of their higher biomass yields (Figure 4B). It was clear however, that addition of ethanol to TM6* cultures did not result in wild-type yields at the corresponding growth phase (Fig 2).

### Functional yields of two human GPCRs and soluble GFP are also increased when produced in TM6* compared with wild-type *S. cerevisiae*

**Figure 5:**
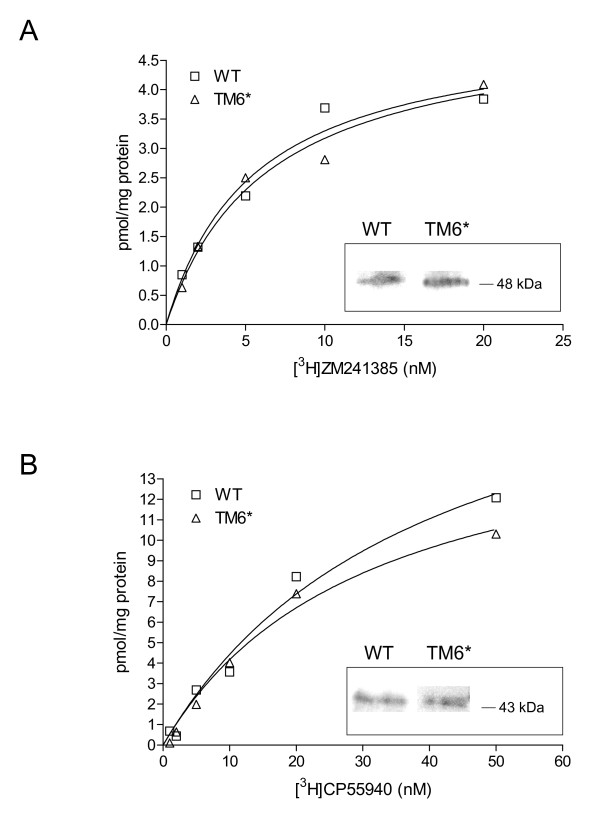
Saturation ligand binding to membrane preparations of wild-type and TM6* cells producing human GPCRs. (A) Labelled and unlabelled ZM241385 ligands were incubated for 60 min at 25 °C with 10 μg cell membrane containing recombinant human A_2a_R. (B) Labelled and unlabelled CP55940 ligands were incubated for 90 min at 30 °C with 10 μg cell membrane containing recombinant human CNR2. Saturation binding curves were analysed by nonlinear regression using Prism Software (GraphPad Software, USA) to calculate B_max _and K_d_. The immunoblot signals for the corresponding proteins are shown as insets within the binding curves.

Having improved the yield of Fps1 in our respiratory *S. cerevisiae *strain, we wished to verify that the yields of other recombinant proteins would also be increased compared to wild-type. We therefore produced the human GPCRs, A_2a_R and CNR2, in TM6* at 30°C (Figure 5). This resulted in a doubling of the wet cell mass in TM6* (9 g and 10 g, respectively; Table 3) compared with wild-type cells (5 g and 4 g, respectively; Table 3) and an increase by at least a factor of 4 in the corresponding total membrane protein yield (5.1 mg and 5.8 mg to 23.3 mg and 29.4 mg, respectively; Table 3). Radioligand binding experiments for A_2a_R yielded respective B_max _and K_d _values of 5.12 (SEM: 0.59) pmol mg^-1 ^and 5.52 (1.68) nM for wild-type membranes compared with 5.16 (0.61) pmol mg^-1 ^and 6.26 (1.86) nM for TM6* membranes. The corresponding values for CNR2 were 22.02 (4.00) pmol mg^-1 ^and 39.75 (12.96) nM for wild-type membranes and 17.02 (2.20) pmol mg^-1 ^and 30.81 (7.71) nM for TM6* membranes. These data illustrate that the yield of recombinant GPCR per unit of total membrane protein is unchanged in the TM6* strain, while the total functional protein yield is at least quadrupled due to an increased biomass yield. Increasing cell biomass as a strategy to increase recombinant protein yield was finally verified in shake-flask cultures for the soluble target, GFP. We found that yields per unit biomass in TM6* were 600 (SEM: 30) mg L^-1 ^OD_610_^-1 ^compared with 500 (20) mg L^-1 ^OD_610_^-1 ^in the wild-type. A doubling of biomass therefore led to a doubling of total volumetric yield.

**Table 3:** Total functional yields from TM6* producing two human GPCRs are at least quadruple that from the corresponding wild-type transformants

Transformants	Duration of culture (h)	Specific growth rate (h^-1^)	Dry cell weight yield (g g^-1 ^Glc)	Harvest wet cell weight (g)	Total membrane protein yield (mg)	B_max _(pmol mg^-1^)	K_d _(nM)
WT CNR2	16	0.3 (0.03)	0.12 (0.02)	5.0	5.1 (0.48)	22.02 (4.00)	39.75 (12.96)
WT A_2a_R	16	0.3 (0.03)	0.12 (0.02)	4.0	5.8 (0.19)	5.12 (0.59)	5.52 (1.68)
TM6* CNR2	25	0.2 (0.02)	0.32 (0.02)	9.0	23.3 (1.00)	17.02 (2.20)	30.81 (7.71)
TM6* A_2a_R	25	0.2 (0.02)	0.32 (0.02)	10	29.4 (0.98)	5.16 (0.61)	6.26 (1.86)

The human GPCRs, A_2a_R and CNR2, were produced in the wild-type *S. cerevisiae *strain, BY4741 (WT), and the respiratory strain, TM6*. Cells were harvested at the time points indicated for preparation of membranes for functional analysis, as described in the Methods section. The mean growth rate (h^-1^) is calculated for the exponential growth phase (n = 2). The mean dry cell weight yield is reported with respect to the glucose consumed (n = 2). Harvest wet cell weight is reported in g, and the mean total membrane protein yield from these cells is reported (n = 3). B_max _and K_d _values were calculated from the curves shown in Figure 5 (n = 3). In all cases the standard error of the mean is given in parentheses.

## Discussion

Structural genomics projects have undertaken the important task of characterising the hundreds of proteins that are encoded by organismal genomes. Most recently, membrane-protein-specific consortia have been established such as E-MeP, MePNet, EDICT and others [34]. Typical activities include high-throughput production of large numbers of membrane proteins, especially GPCRs [35]. Such projects are slowly making important contributions to our understanding of the structural biology of membrane proteins (see http://blanco.biomol.uci.edu/Membrane_Proteins_xtal.html), but by their very nature there is a high attrition rate as individual proteins fail to be produced using high-throughput strategies.

A current and very encouraging trend is to consider membrane protein production as a scientific problem that must be systematically and rationally addressed [12-14]. We and others have contributed to this using yeast [21,22], which has been the production host for a substantial number of recombinant eukaryotic membrane proteins to date. As it is often desirable to obtain structural and functional data on a specific protein, such as a key drug target or an individual member of a biological pathway, this absolutely warrants a systematic, tailored approach. In our own work, we initially focused on the glycerol facilitator from *S. cerevisiae *- Fps1 - on which we have amassed a body of *in vivo *structure-activity data [30,31,36,37]. Despite having constructed a good understanding of the gating mechanism of this intriguing protein, the ability to produce it in amounts sufficient for structural studies has remained elusive [38] (Table 1). In this study we therefore examined production yields in a respiratory *S. cerevisiae *strain, TM6*, with improved biomass-producing properties [29].

We found that the best Fps1 yield from previous studies in wild-type yeast [21] was maintained in the TM6* membrane in cultures grown at 30°C in batch. This is particularly advantageous since the best production conditions are now the optimal growth conditions and hence Fps1 can be produced more quickly in TM6* (generation time 2.9 (SEM: 0.1) h) than in previously-optimised wild-type cultures at 20°C (generation time 8.9 h) [21]. For commercial applications, it is of utmost importance to avoid costs associated with cooling bioreactors and to minimise production times to maximise profit. High-throughput platforms also significantly benefit from shorter generation times. The biggest benefit, however, was the total yield improvement resulting from the improved biomass properties of TM6* compared with the two wild-type strains without the need for complex cultivation regimes. In the glucose phase, maximum yields were achieved in both wild-type strains at an OD_610 _of 4.5 (0.1) during batch-growth with 2 % glucose in the medium. When we produced Fps1 in the respiratory TM6* strain, however, maximum yields were obtained at an OD_610 _of 11.0 (1.5), a 2.4-fold increase in biomass over the wild-type. Consequently, the best yields of Fps1 from TM6* were more than 2-fold higher than the best yields from the wild-type strains. Interestingly, yields of Fps1 from TM6* were not further improved by lowering the temperature, in contrast to wild-type strains where this is routinely observed [21].

We were unable to improve yields of Fps1 in wild-type *S. cerevisiae *by extending the production phase prior to the diauxic shift (Figure 3) or to reduce yields in TM6* by growing cultures in the presence of ethanol, mimicking conditions found with wild-type yeast (Figure 4). We also found that the ethanol concentration was unchanged through the glucose consumption phase (Figure 4A) which indicated that the TM6* cells grew on glucose without co-consuming substantial amounts of ethanol. As soon as the glucose was depleted, the cells started to grow on the ethanol immediately which is seen by the second CO_2 _peak and continued biomass accumulation. We therefore concluded that the improved biomass properties of TM6*, which also enhanced the yields of three additional recombinant proteins, could not be achieved through simple manipulation of culture conditions.

In the related respiratory wine strain V5-TM6*P that we have also generated [39], the genes *BIO2*, *BMS1*, *MSD1 *and *RPO41 *had a changed expression compared with the V5 parent [40] and were also correlated with improved protein yields in a wild-type strain [41]. This further strengthens our earlier observations that *BMS1 *plays a significant role in high yielding recombinant protein production [23]. Interestingly two subunits of the 26S proteosome subunit, *RPT1 *and *RPT2 *are also down regulated in V5-TM6*P compared to V5 by a factor of 0.3 [40]. It is thus possible that the respiratory yeast phenotype has less protease activity than a wild-type strain which in turn negates the need to further slow the process by lowering the temperature.

Overall, it is clear that yeast cells are valuable production hosts on account of their potential for high volumetric yields, short generation times and affordable media formulations, especially in comparison with higher eukaryotic systems. Moreover, the ability to engineer strains with desirable properties that minimise stress responses is straightforward in yeast. In the case of an engineered strain such as TM6*, its improved biomass properties, which result from its respiratory phenotype, make it a particularly valuable resource for challenging proteins such as membrane proteins. For example, we report here the first production of functional human CNR2 in *S. cerevisiae*, and show that we can quadruple its yield by simply switching from a respiro-fermentative to a respiratory strain.

## Conclusions

The TM6* phenotype provides a unique opportunity to improve recombinant protein production yields in *S. cerevisiae *through biomass accumulation. Enhanced volumetric yields were achieved for the yeast glycerol facilitator, Fps1, the human GPCRs, A_2a_R and CNR2, and a soluble GFP. The TM6* respiratory strain should therefore prove to be a useful addition to the matrix of production hosts at the disposal of modern structural biology projects, especially for challenging proteins.

## Methods

### Plasmids

The *FPS1 *gene was tagged at its 3' end with a sequence encoding the HA_3 _epitope to permit immunodetection; the carboxy-terminal threonine residue was replaced with the amino acid sequence: SGRIFYPYDVPDYAGYPYDVPDYAGYPYDVPDYAAQCGR. The HA_3_-tag sequence is underlined. The construct was expressed from the *TPI *promoter in the 2 μ pYX212 vector (Novagen; now discontinued) which contains the *URA3 *selection marker. The gene was cloned into the *Bam*H1 and *Hin*dIII sites and the vector transformed into *S. cerevisiae *TM6*, KOY.PK2-1C82 and BY4741. For the *S. cerevisiae *954 VW K70 strain the construct was expressed from the *TPI *promoter in the 2 *μ *pYX222 vector (Novagen; now discontinued), which contains the *HIS3 *selection marker. Genes encoding human A_2a_R and CNR2 receptors were tagged as above at the 3' end with the HA_3_-tag and at the 5' end with a sequence encoding the *S. cerevisiae *α factor secretion signal: MRFPSIFTAVLFAASSALAAPVNTTTEDETAQIPAEAVIGYSDLEGDFDVAVLPFSNSTNNGLLFINTTIASIAAKEEGVSLEKREAGS. The constructs were expressed from the *TPI *promoter in both the pYX212 and pYX222 vectors. The pCU426 GFP construct was the generous gift of Dr Arle Kruckeberg.

### Yeast strains and growth conditions

The constructs cloned into plasmids pYX212 and pCU426 were transformed into *S. cerevisiae *strains KOY-TM6*, referred to as TM6* [42], KOY.PK2-1C82 (MATa, *ura 3-52*, *MAL 2-8^c ^SUC2*) [43] and BY4741 (MATa; his3Δ1; leu2Δ0; met15Δ0; ura3Δ0). Constructs cloned into plasmid pYX222 were transformed into *S. cerevisiae *strain 954 VW K70 (MATα *his3 SUC2 GAL MAL 2-8^c^*). Yeast transformants were initially grown on YNB agar plates lacking uracil or histidine to select for plasmid retention. For the TM6* Fps1 cultivations, 5 mL cultures were inoculated and grown for 72 h prior to being used to inoculate 50 mL cultures, which were grown for a period of 24 h and used to inoculate cultures in 2.5 L to a final OD_610 _of 0.05. For the wild-type cultivations, 100 mL cultures were grown for 24 h before inoculating the bioreactors to a final OD_610 _of 0.05. Yeast cells were cultured in 2.5 L 2 × CBS medium [44], with 2 % glucose as the sole carbon and energy source. For growth of TM6* with ethanol in the medium, 7 g L^-1 ^ethanol were added to the bioreactor together with glucose just before the inoculation. The pH of the cultures was maintained at pH 5 using 1 M NaOH. Polypropylene glycol P2000 was added as antifoam (100 μL L^-1^). Agitation and aeration of the cultures were set at 1000 rpm and 0.5 vol vol^-1 ^min^-1^, respectively whilst the temperature was set at either 20°C or 30°C. The control of the pH, agitation, aeration and temperature was maintained online. Gas evolution was monitored on-line (type CP460 O_2_/CO_2_, Belach Bioteknik AB).

For glucose fed-batch cultivations, the wild-type *S. cerevisiae *strain 954 VW K70 was cultured at 30°C, pH 5. 100 mL cultures were grown for a period of 24 h and used to inoculate cultures in 2.5 L bioreactors to a final OD_610 _of 0.1. Yeast cells were cultured in 2.0 L YNB medium (1.7 g L^-1 ^YNB (Q-Biogene), 5 g L^-1 ^(NH_4_)_2_SO_4_) with 1 % glucose until glucose was depleted, and then grown with a feed of 500 mL 10 % glucose at a flow rate of 20 mL h^-1^. Polypropylene glycol P2000 was added as antifoam (100 μL L^-1^). Agitation of the cultures was set at 600 rpm and airflow of 0.6 L min^-1^. All cultivations were at least duplicated. We have previously developed a series of simple functional *in vivo *assays for aquaporins in yeast [6] (and references therein), and hence were able to confirm that the Fps1 was active under these conditions. Fps1 activity in wild-type yeast was previously reported [21], and there were no indications of any differences in this study.

BY4741 or TM6* transformed with the GPCR constructs were initially grown on YNB plates lacking uracil to verify plasmid retention. Single colonies were then used to inoculate 50 mL 2 × CBS medium and cultured for a period of 24-48 h. These pre-cultures were then used to inoculate 500 mL 2 × CBS medium in a bioreactor to a final OD_610 _nm of 0.05 and cultured until glucose depletion was detected using a Accu-Chek active glucose analyser (Roche diagnostics, UK) according to the manufacturer's instructions. Cells were harvested by centrifugation at 5, 000 × g, 4°C, 5 min.

BY4741 or TM6* transformed with the GFP construct were grown on YNB plates lacking uracil to verify plasmid retention. Single colonies were then used to inoculate 50 mL YNB medium and cultured for a period of 48 h. These seed cultures were then used to inoculate fresh 50 mL YNB medium in shake-flasks containing 0 or 1 mM CuSO_4 _to a final OD_610 _of 0.15 and cultured for a period of 24 h. Cells were harvested by centrifugation at 5, 000 × g, 4°C, 5 min.

### Protein, cell dry weight and extracellular substrate determination

Samples for optical density measurements were taken periodically to measure growth. For the wild-types and ethanol-phase TM6* cultures, additional samples for subsequent protein, dry weight, and extracellular substrate/product analysis were taken out at early, mid and late glucose phase (1-3-Glc) corresponding to a glucose concentration of 1.5 %, 1.0 % and 0.8 % respectively, 4-Glc (when the glucose is depleted), and 5-EtOH and 6-Stat during the ethanol and stationary phases, 6.5 h, and 18.5 h after glucose depletion, respectively. The same procedure was performed for the TM6* cultures but also at additional time points. Therefore Glc 1-3 correspond to glucose concentrations above 1.5 %, 1.5-0.5 % and below 0.5 %, respectively. 4-Glc corresponds to glucose exhaustion and the stationary phase samples, 5-Stat, for the TM6* cultures are samples taken at least 10 h after glucose exhaustion. For extracellular substrate analysis, samples (2 × 1 mL) were collected, and the supernatant recovered by centrifugation (13, 000 × g for 1 min) and stored at -20°C prior to analysis with Boehringer Mannheim GmbH kits (Food Diagnostics, Stenungsund, Sweden) according to the manufacturer's instructions. Dry weight calculations were performed by harvesting 2 × 5 mL samples in pre-weighed, desiccated sample vials, and the cell pellet recovered by centrifugation at 5,000 × g for 5 min, at 4°C. The cells were subsequently washed twice with 5 mL ice-cold MilliQ water, and recovered by centrifugation, as above. The pellets were then dried for 24 h at 110°C, and stored in a desiccator prior to being weighed. Samples for protein analysis (1-4 × 50 mL) were harvested and the cell pellet recovered by centrifugation at 5,000 × g for 5 min at 4°C and stored at -20°C.

### Membrane preparation and immunoblots

Analysis of Fps1 protein yields were performed on both the total cellular extract and the total membrane fraction. Cells were suspended in 20 mM Tris-HCl (pH 7.6), 100 mM NaCl, 0.5 mM EDTA, 5 % glycerol and mixed with glass beads at a 1:1:1 ratio. The cells were agitated in a FastPrep (Q-Biogene) at a speed of 6.5, 3 × 20 s with a 60 s incubation on ice between the pulses. Unbroken cells were recovered by centrifugation at 500 × g for 10 min at 4°C, and the supernatant further clarified at 10,000 × g for 30 min at 4°C. The total membrane fraction was collected from this clarified supernatant by ultra-centrifugation at 100,000 × g for 90 min at 4°C. The protein concentration was estimated using a BioRad Protein Assay Kit with bovine serum albumin as a standard. Immunoblotting was carried out with 35 μg of crude cell extract and 75 μg of total membrane fraction loaded on a 7.5 % SDS polyacrylamide gel and separated at 65 V through the stacking gel and 140 V through the separating gel for approximately 1 h. Proteins were transferred to a nitrocellulose membrane and blocked with PBS containing 5 % milk for 1 h before being probed with a mouse monoclonal HA antibody (1:1,000) overnight in PBS containing 5 % milk. After washing with PBS containing 5 % milk the membrane was incubated with a secondary goat anti-mouse IgG HRP conjugated antibody (1:2,500) for 1 h in PBS containing 5 % milk. The membrane was then washed in PBS containing 5 % milk; followed by PBS containing 0.1 % Tween-20 and finally with PBS. The blots were developed with an ECL Plus Western Blotting Detection Kit (Amersham Pharmacia) according to the manufacturer's instructions, visualised using the Image Reader LAS-100 (Fujifilm), and quantified using Multi Gauge 3.0 (Fujifilm). Either total extract or the membrane-bound fraction isolated from Sample 2 of a 30°C, pH 5 wild-type culture [21] in YNB medium was used as an internal standard, which is the yield of Fps1 during the glucose growth phase of cells cultured at 30°C, pH 5 when the OD_610 _was 1.5-3.0 and residual glucose levels were 2-5 g/L. All signals were below saturation and related to the signal of the internal standard.

### Radioligand binding on wild-type and TM6* membranes containing recombinant human A_2a_R and CNR2

Cells were washed once with ice-cold breaking buffer (50 mM sodium phosphate buffer pH7.4, 100 mM NaCl, 5 % glycerol, 2 mM EDTA, 1 mM PMSF) and suspended to 30 % wet weight. 0.5 mm glass beads were added to the cell suspension at a 1:1 ratio and cells broken at 4°C using a FastPrep24 cell disrupter (MP Biomedical) with 8 cycles of 30 s shaking and 30 s incubation on ice. Unbroken cells/cell debris were separated from the membrane suspension by centrifugation (3000 × g, 5 min, 4°C). Total membranes were then recovered at 100,000 × g, 45 min, 4°C and suspended in membrane buffer (50 mM Tris pH 8.0, 120 mM NaCl, 20 % glycerol, 1 mM PMSF) using a dounce homogenizer. Membrane proteins were quantified following the BCA method (Pierce, Rockford, IL, USA), using BSA as a standard and snap frozen in liquid nitrogen prior to storage at - 80°C.

Saturation ligand binding assays were performed for membranes containing human A_2a_R with [^3^H]ZM241385 and ZM241385 (10 μM) ligands in 50 mM Tris HCl pH 7.4, 1 mM EDTA, 10 mM MgCl_2_, and incubated at 25°C for 1 h. In the case of human CNR2, [^3^H]CP55940 and CP55940 (50 μM) in 50 mM Tris HCl pH 7.4, 2.5 mM EDTA, 5 mM MgCl_2_, 0.5 mg ml^-1 ^BSA were incubated at 30°C for 1.5 h. For each experiment, 10 μg of membrane protein were incubated in triplicate with increasing concentrations of radioligand until equilibrium was reached (total binding conditions). For non-specific binding determination, similar incubations were performed in parallel in the presence of an excess of unlabelled specific ligand. Bound and free ligands were separated by rapid filtration with Perkin-Elmer GF/B 96-unifilters pre-soaked in 0.3 % polyethylenimine. The filters were washed 3 times and the retained radioactivity measured by liquid scintillation counting in a TopCount scintillation counter (Perkin Elmer). Saturation curves were analysed by nonlinear regression using Prism software (GraphPad Software, La Jolla, USA) to determine B_max _and K_d _values.

### GFP determination in wild-type and TM6* strains

Cells were suspended in 0.7 mL ice-cold breaking buffer (50 mM sodium phosphate buffer pH7.4, 100 mM NaCl, 5 % glycerol, 2 mM EDTA) and glass beads added at a 1:1 ratio. Cells were disrupted in a FastPrep24 for 4 cycles of speed 6.5, 30 s with 2 min incubation on ice between cycles. Cell debris and unbroken cells were removed by centrifugation at 5,000 × g, 4°C for 3 min and the supernatant clarified and recovered at 20,000 × g, 4°C for 30 min. 150 μl of supernatant was mixed with 50 μL 1 M potassium phosphate, pH 8.0 and loaded in triplicate in black Nunc MaxiSorp 96-well plates. The fluorescence was determined on a SpectraMax Gemini XS plate reader (Molecular Devices, Wokingham, UK) with excitation and emission wavelengths of 390 nm and 510 nm respectively and a 495 nm cut-off.

## Competing interests

The authors declare that they have no competing interests.

## Authors' contributions

CF, NB, KH and RD were involved in all aspects of the experimental design, data collection, analysis and interpretation. CL and RW made the GPCR constructs and performed the ligand binding assays. LG and CL contributed to the data analysis and interpretation. RB directed the study, co-ordinated the data analysis and interpretation, and drafted the manuscript. All authors contributed to, read and approved the final version of the manuscript.
